# Norms and equivalences for MoCA-30, MoCA-22, and MMSE in the oldest-old

**DOI:** 10.1007/s40520-021-01886-z

**Published:** 2021-05-29

**Authors:** Zarui A. Melikyan, Michael Malek-Ahmadi, Kathleen O’Connor, Alireza Atri, Claudia H. Kawas, María M. Corrada

**Affiliations:** 1 Institute for Memory Impairments and Neurological Disorders, University of California Irvine, 1511 Hewitt Hall, 843 Health Sciences Road, Irvine, CA 92697 USA; 2 Banner Sun Health Research Institute, Sun City, AZ USA; 3 Center for Brain/Mind Medicine, Department of Neurology, Brigham and Women’s Hospital, Boston, MA USA; 4 Harvard Medical School, Boston, MA USA; 5 Department of Neurology, University of California Irvine, Irvine, CA USA; 6 Department of Neurobiology and Behavior, University of California Irvine, Irvine, CA USA; 7 Department of Epidemiology, University of California Irvine, Irvine, CA USA

**Keywords:** Oldest-old, 90 +, Score conversion, MMSE, MoCA-30, MoCA-22

## Abstract

**Background:**

Cognitive screening is important for the oldest-old (age 90 +). This age group is the fastest growing and has the highest risk of dementia. However, norms and score equivalence for screening tests are lacking for this group.

**Aims:**

To provide norms and score equivalence for commonly used cognitive screening tests for the oldest-old.

**Methods:**

Data on 157 participants of the Center for Healthy Aging Longevity Study aged 90 + were analyzed. First, we derived norms for (1) subtests and cognitive domains of the in-person Montreal Cognitive Assessment having a maximum score of 30 (MoCA-30) and (2) the total MoCA-22 score, obtained from the in-person MoCA-30 by summing the subtests that do not require visual input to a maximum score of 22. These norms were derived from 124 participants with a Mini-Mental State Examination (MMSE) ≥ 27. Second, we derived score equivalences for MMSE to MoCA-30 and MoCA-22, and MoCA-30 to MoCA-22 using equipercentile equating method with log-linear smoothing, based on all 157 participants.

**Results:**

MoCA-22 total score norms are: mean = 18.3(standard deviation = 2.2). An MMSE score of 27 is equivalent to a MoCA-30 score of 22 and a MoCA-22 score of 16.

**Discussion and conclusions:**

Subtest, domain and MoCA-22 norms will aid in evaluation of the oldest-old who cannot complete the MoCA-30 or are tested over the phone. The equivalences of the three cognitive tests (MMSE, MoCA-30, MoCA-22) in the oldest-old will facilitate continuity of cognitive tracking of individuals tested with different tests over time and comparison of the studies that use different cognitive tests.

**Supplementary Information:**

The online version contains supplementary material available at 10.1007/s40520-021-01886-z.

## Introduction

Cognitive screening of the oldest-old (age 90 +) has become increasingly important, because this age group has the highest risk of dementia [1] and its projected growth in the coming decades is rapid [2]. However, cognitive testing of this age group is challenging. First, sensory and cognitive impairments make many of the oldest-old unable to complete all subtests of in-person screening measures, which makes calculation of the total score and its comparison to normative values impossible. In such situations, subtest and domain norms allow for evaluation of completed subtests. In that vein, subtest and domain norms in younger-old (older adults younger than 90 years) have been published for one of the most frequently used screening measures, the in-person Montreal Cognitive Assessment that has a maximum possible score of 30 (MoCA-30) [3–6]. Additionally, MoCA-Blind [7], that includes MoCA-30 subtests that do not require visual input and has a maximum possible score of 22, was developed to enable in-person cognitive screening of individuals with visual impairment. MoCA-Blind normative cut-points, to distinguish cognitively normal from cognitively impaired younger-old, were published for the sum of in-person MoCA-30 subtests included in MoCA-Blind [8]. Henceforth, the sum of in-person MoCA-30 subtests included in MoCA-Blind, that has maximum possible score of 22, is called MoCA-22. The second challenge in testing the oldest-old is that prevalent frailty and other comorbidities prevent many of them from travelling to testing sites, which makes telephone screening a method of choice. To address the need for telephone cognitive screening, Telephone MoCA [7], identical to MoCA-Blind with slightly modified testing procedures to accommodate telephone testing, was developed and normative cut-points published for younger-old [9–12]. However, no norms for MoCA-30 subtests and domains or MoCA-Blind/ Telephone MoCA total score have been published for the oldest-old.

Recently, many clinical and research settings have switched to using the MoCA-30/ MoCA-Blind/ Telephone MoCA from the Mini-Mental State Exam (MMSE) [13], another common screening measure. The MoCA-30 is more sensitive to mild forms of cognitive impairment, has a higher diagnostic accuracy than the MMSE [14–17], and is available at no cost. To facilitate continuity of cognitive tracking in individuals tested with MMSE, MoCA-30, and MoCA-Blind/ Telephone MoCA at different times and to provide comparability of data among multiple studies and trials, the ability to equate scores of these three tests is necessary. Equating MMSE to MoCA-30 in the younger-old shows that, although these two tests have identical score range (0–30), they do not have one-to-one correspondence. Rather, higher MMSE scores correspond to lower MoCA-30 scores, likely because some of MoCA-30 subtests are more challenging [16, 18, 19]. While two studies have equated MMSE and MoCA-30 to Telephone MoCA and MoCA-22 in the younger-old [19, 20], equivalencies for the oldest-old have not been reported. Lacking Telephone MoCA data, and given that equivalence of Telephone MoCA and MoCA-22 was recently demonstrated [20], we used the MoCA-22 as the next best option to equate it with the MMSE and MoCA-30.

The aims of this study are to provide in the oldest-old: (1) norms for subtests and cognitive domains of in-person MoCA-30 and norms for MoCA-22 total score derived from in-person MoCA-30 by summing its subtests that do not require visual input, and (2) score equivalence of MMSE to MoCA-30 and MoCA-22, and MoCA-30 to MoCA-22.

### Methods

### Study procedures

We report on a subset of participants of the ongoing Center for Healthy Aging Longevity Study [21]. Since its inception in 2007, the study has collected demographic, physical, and cognitive data on older adults aged 50–110 years to explore factors associated with increased longevity and healthy aging. Study enrollment occurs continuously and participants are recruited through advertisements, community talks, referrals from current study participants throughout the state of Arizona, predominantly in Sun Cities of the northwest Phoenix metropolitan area. All participants are community-dwelling, independent individuals deemed to be cognitively unimpaired based on structured interviews, testing, and study protocol assessment, and supported by self-reported medical history of no dementia, and no cognitive, neurological or other diagnoses with high likelihood to cause cognitive impairment. All individuals reported independence in personal, home and community affairs and no difficulties or support for activities of daily living that related to cognition. Participants are assessed annually either at the Banner Sun Health Research Institute or at their residences. Cognitive diagnosis is not assigned as part of the research visit.

The study was approved by the Western Institutional Review Board and all participants provided signed informed consent. Research was completed in accordance with the Helsinki Declaration.

### Participants and data items

We requested from the Center for Healthy Aging Longevity Study data on participants who were aged 90 or older and who had completed both MoCA-30 and MMSE in-person at their first cognitive testing. We requested MoCA-30 (version 7.1) [7] total and subtest scores, MMSE total scores, and total score for the Center for Epidemiologic Studies Depression Scale (CES-D) [24]. There were no missing scores in the data provided. In addition, we requested information on sex, race, age at first visit, residence type (own housing, independent living facility with congregate meals, assisted living facility, nursing home or care center), and education. Health information was not available. Data on 157 eligible participants were provided on Feb 27, 2020.

Normative data were derived for a subgroup of 124 participants with MMSE ≥ 27, a cut point that differentiates normal cognition from mild cognitive impairment and dementia [22, 23]. Data from the 157 participants were used to provide score equivalence of MMSE to MoCA-30 and MoCA-22, and MoCA-30 to MoCA-22.

To obtain the total MoCA-22 score, we summed the scores for MoCA-30 subtests that that do not require visual input (i.e., digit span forward, digit span backwards, letter A tapping, serial 7s, sentence repetition, letter F fluency, similarities, delayed recall, orientation to time and place), as was done in previous publications [19, 20] (Table 1). One point was added to total MoCA-30 and MoCA-22 scores for individuals with ≤ 12 years of education according to the manual.

**Table 1:** Cognitive domains and subtests included in MoCA-30 and MoCA-22

Cognitive domain	Subtest	MoCA-30	MoCA-22
Visuospatial/Executive	Trail Making Test	+	
	Copy cube	+	
	Draw clock	+	
Naming	Picture naming	+	
Attention	Digit Span forward	+	+
	Digit Span backwards	+	+
	Letter A tapping	+	+
	Serial 7s	+	+
Language	Sentence repetition	+	+
	Letter F fluency	+	+
Abstraction	Similarities	+	+
Memory	Delayed recall	+	+
Orientation	Orientation to time and place	+	+

*MoCA-30*  Montreal Cognitive Assessment administered in-person with a maximum possible score of 30, *MoCA-22*  Version of MoCA-30 that excludes items that require visual input and has a maximum possible score of 22

“ + ” means the subtest is included in the MoCA-30 or MoCA-22

### Data analysis

To provide norms for MoCA-30 domains and subtests and for MoCA-22 total score, we used a subgroup of participants with MMSE ≥ 27 (the normative subgroup). Norms are reported as means, standard deviations, and percent of participants who obtained the highest possible domain or subtest score. For example, the maximum score for visuospatial/executive domain is five and we provide the percent of participants from the normative subgroup who obtained a score of five on this domain. Subtest norms are reported as the percent of participants that obtained highest possible score on each subtest. For example, maximum score on the Trail Making subtest is one and we provide the percent of participants from the normative subgroup who obtained a score of one on this subtest. Norms are provided for the following age categories: 90–91, 92–94, and ≥ 95 years, which were chosen because they contain approximately equal number of participants and correspond to our previous normative publications [25, 26]. The effect of age group on the scores was assessed by Kruskal–Wallis test.

To derive equivalence of MMSE to MoCA-30 and MoCA-22, and MoCA-30 to MoCA-22 total scores, we used the equipercentile equating method with log-linear smoothing [16–19, 27–30]. This method equates scores from two measures based on the equivalency of their corresponding percentile ranks [31]. The log-linear smoothing of the raw scores before the equipercentile equating is used, because raw score distributions are often irregular, i.e., the percentage of the test takers with a given score does not change gradually as the score increases or decreases. These irregularities produce irregularities in equipercentile equating adjustment and those irregularities, in turn, do not generalize to other groups of test takers. The log-linear smoothing technique mitigates this issue by replacing the observed score distribution with a distribution that has the same location, spread and shape, but not the irregularities.

Demographic data are expressed as means, standard deviations, and ranges for numeric variables, and percentage and frequencies for the categorical variables.

All analyses were done using R-Studio version 1.1.414 with R version 3.4.3. [32]. Log-linear smoothing and equipercentile equating were done using the ‘equate’ R library [33].

## Results

### Characteristics of the study groups: demographics and total cognitive test scores

The entire group of 157 participants had a mean age of 93.5 years (range: 90–102). Most participants were women (67%), well-educated (mean years = 15.1), and lived independently (94%). All participants were White (Table 2).

**Table 2:** Characteristics of study participants for the entire group and the normative subgroup defined as MMSE ≥ 27

Characteristic	Entire group (*N* = 157)	Normative subgroup (*N* = 124)
	*N* (%)	
Sex, women	105 (66.9)	90 (72.6)
Age group, years 90–91 92–94 ≥ 95	45 (28.7) 57 (36.3) 55 (35.0)	41 (33.1) 43 (34.7) 40 (32.3)
Race, White	157 (100)	124 (100)
Education level, > high school	120 (76.9)	95 (77.2)
Residence type Own residence Independent living with congregate meals Assisted living, nursing home or care center	92 (58.6) 55 (35.0) 9 (5.7)	77 (62.1) 41 (33.1) 5 (4.0)

	Mean (SD) [Range]	
Age, years	93.5 (2.9) [90–102]	93.5 (2.9) [90–101]
Education, years	15.1 (1.8) [9–18]	15.3 (1.7) [9–18]
MoCA-30	24.2 (2.8) [17–30]	24.8 (2.5) [19–30]
MoCA-22	17.7 (2.4) [11–22]	18.3 (2.2) [13–22]
MMSE	28.1 (1.8) [21–30]	28.8 (1.0) [27–30]
CES-D	4.9 (4.8) [0–27]	4.7 (4.7) [0–27]

*MoCA-30*  Montreal Cognitive Assessment administered in-person with a maximum possible score of 30

*MoCA-22*  Version of MoCA-30 that excludes items that require visual input and has a maximum possible score of 22

*MMSE*  Mini-Mental State Examination, *CES-D* Center for Epidemiologic Studies Depression Scale

The demographic characteristics of the normative subgroup of 124 participants with MMSE ≥ 27 were comparable to those of the entire group. In the normative subgroup, the mean age was 93.5 years (range: 90–101); most were women (73%), well-educated (mean years = 15.3), and lived independently (96%). All the normative group participants were White (Table 2).

In the entire group of 157 participants the mean MoCA-30 score was 24.2, the mean MoCA-22 score was 17.7, and the mean MMSE score was 28.1. In the normative subgroup, the mean MoCA-30 score was 24.8, the mean MoCA-22 score 18.3, and the mean MMSE score was 28.8, each about 0.7 higher than in the entire group (Table 2). Mean total MoCA-30, MoCA-22, and MMSE scores did not significantly differ by residence type in the entire group or in the normative subgroup (Table 3).

**Table 3:** Mean (standard deviation) of cognitive test scores by place of residence

		Own residence	Independent living with congregate meals	Assisted living, nursing home or care center	*p* value
Entire group	MoCA-30	24.4 (2.6)	24.0 (2.8)	22.1 (3.3)	0.18
	MoCA-22	18.0 (2.3)	17.4 (2.5)	15.7 (2.9)	0.06
	MMSE	28.3 (1.6)	27.9 (2.1)	26.9 (2.0)	0.19
Normative subgroup	MoCA-30	24.8 (2.5)	24.9 (2.5)	23.6 (2.7)	0.47
	MoCA-22	18.3 (2.2)	18.2 (2.3)	17.0 (2.9)	0.48
	MMSE	28.8 (1.1)	29.0 (0.9)	28.4 (0.9)	0.57

*MoCA-30*  Montreal Cognitive Assessment administered in-person with a maximum possible score of 30;

*MoCA-22*  Version of MoCA-30 that excludes items that require visual input and has a maximum possible score of 22;

*MMSE*  Mini-Mental State Examination;

*p* value for difference among residential groups from Kruskal–Wallis test

### Norms for MoCA-30 subtest and domain scores and MoCA-22 total score

The percent of participants with the highest possible domain scores was lowest for memory (15% of participants) and visuospatial/executive (25% of participants). Conversely, the percent was highest for the domains of orientation (89% of participants) and naming (78% of participants) (Table 4). The MoCA-22 normative group mean total score is 18.3 and the standard deviation is 2.2. The Supplement contains norms for each domain by age category and overall (Supplementary Table 1), and percent of participants with highest subtest scores by age category and overall (Supplementary Table 2).

**Table 4:** Mean, standard deviation (SD), and range of total cognitive test and domain scores for MoCA-30 and MoCA-22 for the normative subgroup defined as MMSE ≥ 27

Test/domain (possible score range)	Mean	SD	Actual score range	Percent of participants with the highest possible total or domain score
MoCA-30 (0–30)	24.8	2.5	19–30	2
Visuospatial/executive (0–5)	3.8	1.1	0–5	25
Naming (0–3)	2.8	0.4	2–3	78
Attention (0–6)	5.5	0.8	2–6	67
Language (0–3)	2.4	0.7	1–3	53
Abstraction (0–2)	1.7	0.6	0–2	77
Memory (0–5)	2.6	1.7	0–5	15
Orientation (0–6)	5.8	0.6	0–6	89
MoCA-22 (0-22)	18.3	2.2	13–22	6

*MoCA-30*  Montreal Cognitive Assessment administered in-person with a maximum possible score of 30;

*MoCA-22* Version of MoCA-30 that excludes items that require visual input and has a maximum possible score of 22

### Equivalences of MMSE, MoCA-30, and MoCA-22 Scores

**Fig. 1:**
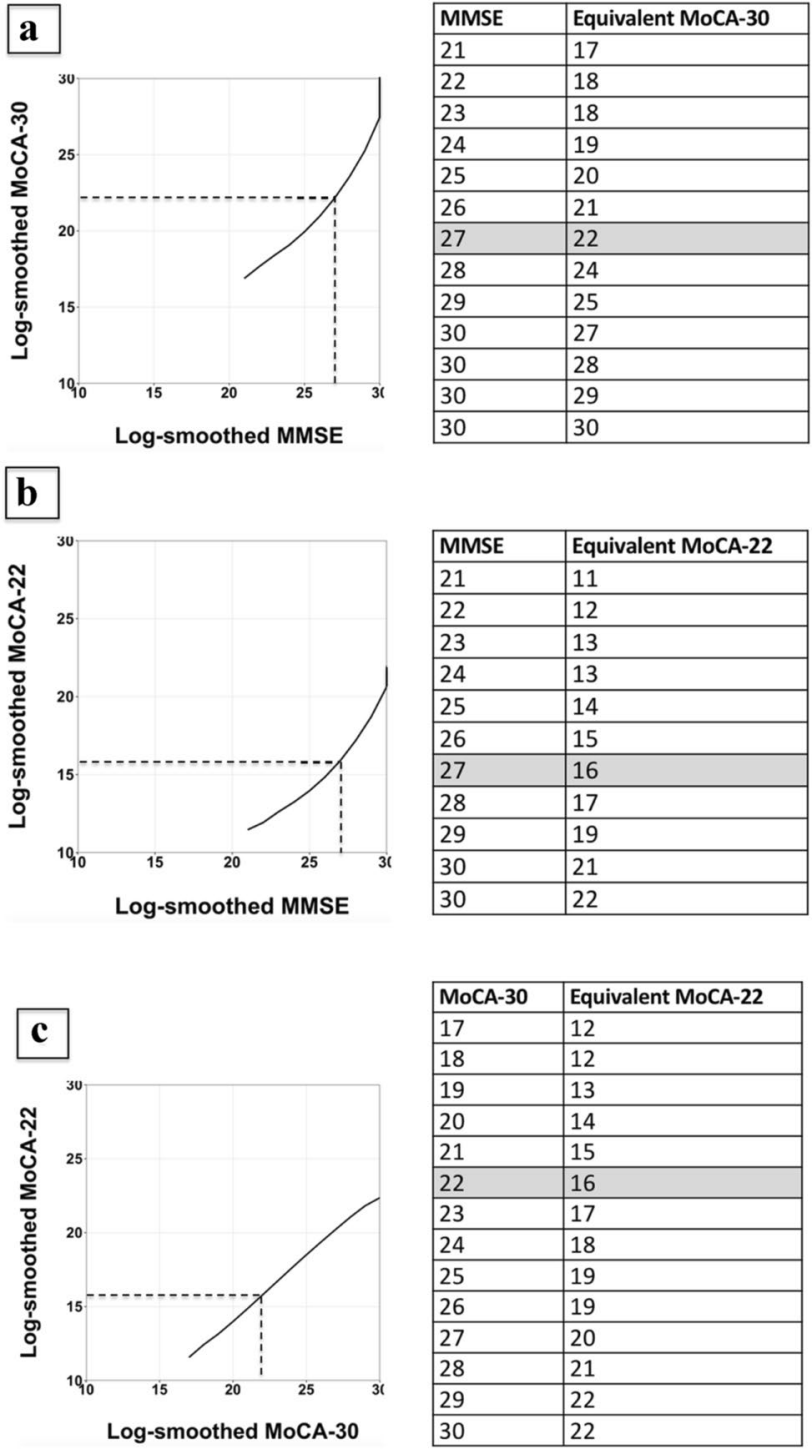
**a** Mini-Mental State Examination (MMSE) mapped to Montreal Cognitive Assessment with a maximum possible score of 30 (MoCA-30) using equipercentile equating method. Dotted lines indicate that 27 points on the MMSE is equivalent to 22 points on the MoCA-30. These scores are also shaded on the table. **b** MMSE mapped to Montreal Cognitive Assessment with a maximum possible score of 22 (MoCA-22) using equipercentile equating method. Dotted lines indicate that 27 points on the MMSE is equivalent to 16 points on the MoCA-22. These scores are also shaded on the table. **c** MoCA-30 mapped to MoCA-22 using equipercentile equating method. Dotted lines indicate that 22 points on MoCA-30 is equivalent to 16 points on the MoCA-22. These scores are also shaded on the table

The equivalences of MMSE to MoCA-30 and MoCA-22, and MoCA-30 to MoCA-22 scores using the equipercentile equating method with log-linear smoothing are shown in Fig. 1. An MMSE score of 27 was equivalent to a MoCA-30 score of 22 and to a MoCA-22 score of 16. A MoCA-30 score of 22 is equivalent to a MoCA-22 score of 16.

## Discussion

This work provides norms for MoCA-30 subtests and domains, and MoCA-22 total score derived from oldest-old adults with normal cognition defined as MMSE ≥ 27. These norms allow interpretation of MoCA-30 performance for oldest-old individuals who cannot complete the entire test or were tested over the telephone. This study also provides in the oldest-old score equivalences of the three tests (MMSE, MoCA-30, MoCA-22) to facilitate comparison and conversion of scores between multiple centers and trials and within studies that used different tests at different times. The norms and score equivalences of these three tests provided in this paper are important in facilitating the use of screening measures in the oldest-old.

While both MMSE and MoCA-30 were administered in-person to participants in this study, MoCA-22 was not administered as a separate instrument. It was derived from the scores of the in-person MoCA-30 by summing those subtests that do not require visual input. Previous studies have demonstrated equivalence between the Telephone MoCA and the MoCA-22 [20], and similar reliability (area under the ROC curve) of these two measures to detect mild cognitive impairment [10]. Our results are particularly useful for evaluations that use Telephone MoCA or MoCA-Blind, given the absence of any information on these tests in the oldest-old.

Our total and domain norms for MoCA-30 and MoCA-22 total score generally correspond well to the two studies that published similar norms, albeit in younger-old [4, 5] (see Supplementary table 3 summarizing our norms and norms from these two studies). However, total MoCA-30 score and scores on visual-spatial/executive and memory domains are lower in our study than in the 1 study of 758 members of Swedish population-based cohort age 65–85 years [4]. This difference is likely due to younger age and perhaps to the more rigorous procedure to determine cognitive status in the Swedish study. A third study that provides percent of cognitively normal older adults with the highest score on each MoCA-30 subtest also reports results that are similar to ours [3]. In our study, domain and subtest norms do not differ significantly by age group (Supplementary Tables 1, 2). Given that age was used as categorical variable and that subtests often had narrow score ranges (e.g., some subtests had only two possible scores: 0 or 1), the study does not have the precision to observe the a priori expected age-related decrements in performance on all the tests. Until there is additional data to further delineate performance in the oldest-old, we suggest using total, rather than the age-group-specific, subtest scores as a normative reference.

We determined the equivalence of MMSE to MoCA-30 and MoCA-22, and MoCA-30 to MoCA-22 scores using the equipercentile equating method as was done in most studies [16–20, 27–30]. We found that an MMSE score of 27 corresponds to a MoCA-30 score of 22, which is similar to other reports where MMSE score of 27 is equivalent to MoCA-30 scores of 21–24 [16–19, 27–30, 34–37]. These studies (reviewed in Supplementary Table 4) included younger-old participants with cognition ranging from normal to dementia.

The MMSE and MoCA-30 are both global cognitive screening measures with the same range of scores (0–30), so it might be erroneously assumed that their scores have a one-to-one correspondence. However, the two tests emphasize different aspects of cognition. While the MMSE allocates more points on orientation (10 out of 30) than the MoCA-30 (6 out of 30), the MoCA-30 places a greater emphasis on visuospatial domain (5 out of 30) than the MMSE (1 out of 30). As a consequence, it is not surprising that these two tests do not have a strictly linear relationship as demonstrated here and supported by previous evidence [18]. Also, some subtests in the MoCA-30 may be more challenging than those in the MMSE (e.g., delayed recall on the MoCA-30 involves five words with a longer delay compared to the three words and shorter delay in MMSE), which contributes to lower MoCA-30 scores as reported here and in previous publications [16–18, 27, 36].

In this study, an MMSE score of 27 equates to a MoCA-22 score of 16. The only relevant publication we found equates MMSE score of 27 to MoCA-22 score of 17 (derived from MoCA-30) in a younger (mean age = 84 years) group of 119 patients from general and geriatric medicine [19]. To put our finding in additional context, a MoCA-22 score of 16, which could be suggested as a cut point for cognitive impairment because it corresponds to an MMSE cut point for cognitive impairment, is lower but generally comparable to the MoCA-Blind cut point of 18 or 19 suggested by the test author and validated in younger ages (55–85 years) [38], and to the Telephone MoCA cut-points of 17–19 suggested for younger-old (age range: 62–75 years) [8–11] (also reviewed in Supplementary table 5). We demonstrated that a MoCA-30 score of 22 equates to a MoCA-22 score of 16, which is the same as that in a study of younger-old that equated the in-person MoCA-30 to the Telephone MoCA [20]. Similarly, in another study of younger-old, a MoCA-30 score of 22 equates to a MoCA-22 score of 17 derived from the in-person MoCA-30 [19].

This paper has several notable strengths. First, we report data on a relatively large sample of individuals aged 90 + . This allowed us to investigate the age effect on the test scores in the oldest-old. Second, we use equipercentile equating method that was previously used with younger-old and our results are comparable with those studies. Third, we report on widely used cognitive screening tests, MoCA-30 and MMSE. Fourth, normative data on screening subtests and domains for the oldest-old is reported for the first time.

We acknowledge several limitations. First, our sample represents mostly the upper part of the cognitive spectrum in the oldest-old; thus, we were not able to provide score equivalences for the lower functioning individuals. However, the high cognitive status of our participants makes our study comparable to others, which also have high prevalence of the upper spectrum of cognitive scores [28], and the agreement between our and other studies [3–5, 16–20, 30, 34] indicates high reliability of our findings. Second, generalizability of our findings to different racial and educational groups is limited as our sample is all White (compared to 88% in oldest-old in the U.S.) and highly educated (77% in our study vs. 28% in the oldest-old in the U.S. have more than a high school education). At the same time, our sample of about two-thirds women, closely matches the sex distribution of the oldest-old in the U.S. (67% in our group vs. 70% in oldest-old in the U.S.) [39]. Although norms derived from diverse populations are needed, in the absence of such norms for the oldest-old, these norms may be used with caution keeping in mind differential effect of health, educational and other factors that affect test performance in diverse populations [40]. Third, both MMSE and MoCA-30 were administered on the same day, with MMSE being administered before MoCA-30. The order of administration may have altered MoCA-30 performance either through learning effects for similar items or mental fatigue. Fourth, although studies show that test performance is related to health status, data on the health characteristics of our participants were not available to us. Fifth, although sample sizes for age subgroup norms are modest (about 40 per group), they are close to a desirable size of 50 that provides a stable estimate of population mean [41].

## Conclusions

MoCA-30 domain and subtest norms and MoCA-22 norms for the oldest-old provided in this paper will facilitate evaluation of oldest-old individuals who are unable to complete the entire MoCA-30 or who are tested over the telephone. Score equivalences of MMSE, MoCA-30 and MoCA-22 in the oldest-old provided in this paper will help maintain continuity of care when multiple evaluations use different tests at different times and will allow data comparability among different studies.

## Supplementary Information

Below is the link to the electronic supplementary material.

Supplementary file1 (DOCX 40 kb)

## Data Availability

The dataset used for the current study is available from the corresponding author on reasonable request.

## Code availability

The R code used for the current study is available from the corresponding author on reasonable request.
