# Supplementary material for: Norms and equivalences for MoCA-30, MoCA-22, and MMSE in the oldest-old
Source: Aging Clin Exp Res. 2021 May 29;33(12):3303–11. doi: 10.1007/s40520-021-01886-z (PMC8668848; doi:10.1007/s40520-021-01886-z)
Supplement: Supplementary file 1 — Supplementary file1 (DOCX 40 kb) [file 40520_2021_1886_MOESM1_ESM.docx]

**Supplement**

**Journal: Aging Clinical and Experimental Research**

**Norms and equivalences for MoCA-30, MoCA-22, and MMSE in the oldest-old**

Zarui A. Melikyan, Michael Malek-Ahmadi, Kathleen O’Connor, Alireza Atri, Claudia H. Kawas, Maria M. Corrada

Corresponding author:

Zarui A. Melikyan, Ph.D.

Institute for Memory Impairments and Neurological Disorders, University of California, Irvine, CA, USA

Email: [zmelikya@uci.edu](mailto:zmelikya@uci.edu)

| **Supplementary Table 1** MoCA-30 total and cognitive domain scores, and MoCA-22 total score for the normative subgroup, defined as MMSE≥27, as a whole and by age | | | | | | |
| --- | --- | --- | --- | --- | --- | --- |
| Domain / Test (Possible score range) | Age group* | Mean | SD | Actual score range | KWχ^2^,  *p*-value† | Percent of participants with the highest possible total or domain score |
| MoCA-30 (0-30) | 90-91 | 24.6 | 2.6 | 19-30 | 5.07, 0.08 | 2 |
|  | 92-94 | 25.4 | 2.3 | 20-29 |  | 0 |
|  | ≥95 | 24.3 | 2.6 | 20-29 |  | 0 |
|  | Overall | 24.8 | 2.5 | 19-30 |  | 2 |
| Visuospatial/ Executive (0-5) |  |  |  |  |  |  |
|  | 90-91 | 3.7 | 1.3 | 0-5 | 0.27, 0.87 | 29 |
|  | 92-94 | 3.8 | 1.0 | 0-5 |  | 21 |
|  | ≥95 | 3.8 | 1.0 | 2-5 |  | 28 |
|  | Overall | 3.8 | 1.1 | 0-5 |  | 25 |
| Naming (0-3) |  |  |  |  |  |  |
|  | 90-91 | 2.8 | 0.4 | 2-3 | 2.45, 0.29 | 80 |
|  | 92-94 | 2.8 | 0.4 | 2-3 |  | 84 |
|  | ≥95 | 2.7 | 0.5 | 2-3 |  | 70 |
|  | Overall | 2.8 | 0.4 | 2-3 |  | 78 |
| Attention (0-6) |  |  |  |  |  |  |
|  | 90-91 | 5.3 | 0.9 | 2-6 | 4.82, 0.09 | 54 |
|  | 92-94 | 5.6 | 0.8 | 3-6 |  | 72 |
|  | ≥95 | 5.7 | 0.6 | 4-6 |  | 75 |
|  | Overall | 5.5 | 0.8 | 2-6 |  | 67 |
| Language (0-3) |  |  |  |  |  |  |
|  | 90-91 | 2.6 | 0.5 | 1-3 | 5.61, 0.06 | 63 |
|  | 92-94 | 2.5 | 0.6 | 1-3 |  | 53 |
|  | ≥95 | 2.2 | 0.8 | 1-3 |  | 43 |
|  | Overall | 2.4 | 0.7 | 1-3 |  | 53 |
| Abstraction (0-2) |  |  |  |  |  |  |
|  | 90-91 | 1.7 | 0.6 | 0-2 | 1.79, 0.41 | 78 |
|  | 92-94 | 1.8 | 0.5 | 0-2 |  | 81 |
|  | ≥95 | 1.6 | 0.7 | 0-2 |  | 70 |
|  | Overall | 1.7 | 0.6 | 0-2 |  | 77 |
| Delayed recall (0-5) |  |  |  |  |  |  |
|  | 90-91 | 2.4 | 1.8 | 0-5 | 4.67, 0.09 | 17 |
|  | 92-94 | 3.0 | 1.6 | 0-5 |  | 16 |
|  | ≥95 | 2.2 | 1.7 | 0-5 |  | 10 |
|  | Overall | 2.6 | 1.7 | 0-5 |  | 15 |
| Orientation (0-6) |  |  |  |  |  |  |
|  | 90-91 | 5.9 | 0.3 | 5-6 | 1.71, 0.43 | 90 |
|  | 92-94 | 5.7 | 1.0 | 0-6 |  | 84 |
|  | ≥95 | 5.9 | 0.4 | 4-6 |  | 93 |
|  | Overall | 5.8 | 0.6 | 0-6 |  | 89 |
| MoCA-22 (0-22) |  |  |  |  |  |  |
|  | 90-91 | 18.1 | 2.3 | 14-22 | 4.98, 0.08 | 2 |
|  | 92-94 | 18.8 | 2.3 | 13-22 |  | 3 |
|  | ≥95 | 17.8 | 2.1 | 14-22 |  | 1 |
|  | Overall | 18.3 | 2.2 | 13-22 |  | 6 |
| **^*^**In years. **^†^**Kruskal-Wallis chi-square and *p*-value for comparing the mean ranks of sub-test scores among the age groups.  MoCA-30=Montreal Cognitive Assessment administered in-person with a maximum possible score of 30;  MoCA-22=Version of MoCA-30 that excludes items that require visual input and has a maximum possible score of 22. | | | | | | |

| **Supplementary Table 2** Percent of participants with the highest possible score on each MoCA-30 subtest for the normative subgroup, defined as MMSE≥27, as a whole and by age | | |
| --- | --- | --- |
| Age group, years | Percent of participants with the highest possible score | |
|  | Trail Making Test | Letter F fluency |
| 90-91 | 56 | 78 |
| 92-94 | 67 | 70 |
| ≥95 | 75 | 70 |
| Overall | 66 | 73 |
|  | Cube copy | Abstraction 1 |
| 90-91 | 63 | 88 |
| 92-94 | 63 | 91 |
| ≥95 | 45 | 75 |
| Overall | 57 | 85 |
|  | Clock contour | Abstraction 2 |
| 90-91 | 95 | 83 |
| 92-94 | 98 | 86 |
| ≥95 | 98 | 82 |
| Overall | 97 | 84 |
|  | Clock numbers | Delayed recall 1 |
| 90-91 | 88 | 49 |
| 92-94 | 86 | 56 |
| ≥95 | 92 | 42 |
| Overall | 89 | 49 |
|  | Clock hands | Delayed recall 2 |
| 90-91 | 68 | 61 |
| 92-94 | 65 | 74 |
| ≥95 | 65 | 57 |
| Overall | 66 | 65 |
|  | Naming picture 1 | Delayed recall 3 |
| 90-91 | 100 | 46 |
| 92-94 | 98 | 63 |
| ≥95 | 95 | 40 |
| Overall | 98 | 50 |
|  | Naming picture 2 | Delayed recall 4 |
| 90-91 | 80 | 29 |
| 92-94 | 86 | 51 |
| ≥95 | 78 | 38 |
| Overall | 81 | 40 |
|  | Naming picture 3 | Delayed recall 5 |
| 90-91 | 100 | 56 |
| 92-94 | 100 | 56 |
| ≥95 | 98 | 42 |
| Overall | 99 | 52 |
|  | Digit Span Forward | Date |
| 90-91 | 85 | 93 |
| 92-94 | 98 | 86 |
| ≥95 | 95 | 95 |
| Overall | 93 | 91 |
|  | Digit Span Backwards | Month |
| 90-91 | 85 | 98 |
| 92-94 | 95 | 98 |
| ≥95 | 90 | 98 |
| Overall | 90 | 98 |
|  | Letter A | Year |
| 90-91 | 100 | 100 |
| 92-94 | 98 | 98 |
| ≥95 | 100 | 100 |
| Overall | 99 | 99 |
|  | Serial 7s | Day |
| 90-91 | 71 | 100 |
| 92-94 | 79 | 98 |
| ≥95 | 85 | 98 |
| Overall | 78 | 98 |
|  | Sentence 1 | Place |
| 90-91 | 98 | 100 |
| 92-94 | 95 | 98 |
| ≥95 | 82 | 100 |
| Overall | 92 | 99 |
|  | Sentence 2 | City |
| 90-91 | 85 | 100 |
| 92-94 | 81 | 95 |
| ≥95 | 70 | 100 |
| Overall | 79 | 98 |
| MoCA-30=Montreal Cognitive Assessment administered in-person with a maximum possible score of 30. | | |

| **Supplementary table 3** Comparison of MoCA-30 total and cognitive domain scores, MoCA-22 total score in the present and previous work | | | | | | |
| --- | --- | --- | --- | --- | --- | --- |
| Present work | | | | | Borland, [4] | Cesar, [5] |
| Test / Domain  (Possible range of scores) | Mean | SD | Actual range of scores | Percent of participants with the highest possible total or domain score | Mean(SD) | Mean(SD) |
| MoCA-30 (0-30) | 24.8 | 2.5 | 19-30 | 2 | 26.0(2.3) | 23.9(3.1) |
|  |  |  |  |  |  |  |
| Visuospatial/ Executive (0-5) | 3.8 | 1.1 | 0-5 | 25 | 4.1(1.0) | 3.9(1.2) |
|  |  |  |  |  |  |  |
| Naming (0-3) | 2.8 | 0.4 | 2-3 | 78 | 2.9(0.3) | 2.7(0.5) |
|  |  |  |  |  |  |  |
| Attention (0-6) | 5.5 | 0.8 | 2-6 | 67 | 5.7(0.8) | 5.5(0.8) |
|  |  |  |  |  |  |  |
| Language (0-3) | 2.4 | 0.7 | 1-3 | 53 | 2.6(0.9) | 2.3(0.7) |
|  |  |  |  |  |  |  |
| Abstraction (0-2) | 1.7 | 0.6 | 0-2 | 77 | 1.7(0.6) | 1.5(0.8) |
|  |  |  |  |  |  |  |
| Delayed recall (0-5) | 2.6 | 1.7 | 0-5 | 15 | 3.1(1.3) | 2.1(1.6) |
|  |  |  |  |  |  |  |
| Orientation (0-6) | 5.8 | 0.6 | 0-6 | 89 | 6.0(0.2) | 5.9(0.2) |
|  |  |  |  |  |  |  |
| MoCA-22 (0-22) | 18.3 | 2.2 | 13-22 | 6 | - | - |
| MoCA-30=Montreal Cognitive Assessment administered in-person with a maximum possible score of 30;  MoCA-22=Version of MoCA-30 that excludes items that require visual input and has a maximum possible score of 22. | | | | | | |

| **Supplemental table 4** Studies that equate MMSE to MoCA-30 scores in older adults | | | | | | | | | |
| --- | --- | --- | --- | --- | --- | --- | --- | --- | --- |
| First author, [reference] | Cohort and country | Diagnoses | Sample size | Age: mean±SD (range) | MMSE mean±SD (range) | MoCA-30 mean±SD (range) | MoCA-30 score adjusted for education | MoCA-30 equivalent to MMSE of 27 | Score equating method |
| Bergeron, [28] | Academic memory clinics, Canada | AD, MCI, VaD, FTD, PPA, PDD, corticobasal syndrome, PSP, psychistric disorders, subjective memory complaints | 1,492 | 69±11 (NR) | NR  (20.5-30) | NR  (10-28) | No | 21 | Equipercentile equating with log-linear smoothing |
| Dharmasaroja, [34] | Multicenter Dementia Registry, Thailand | CN, MCI, dementia | 183 | 68.3±9.4 (NR) | 24.9±2.9 (NR) | 19.1±3.4 (NR) | NR | 21, 22 | Linear regression |
| Helmi, [18] | Psychiatry outpatient clinic and hospital, Ireland | Psychiatric disorders | 70 | 77.36±7.06 (62-89) | 24.47±4.87 (9-30) | 19.03±6.35 (4-29) | NR | 21, 22 | Circle-Arc method |
| Larner, [35] | Regional Neuroscience Center, UK | MCI, dementia | 150 | 20-87 (NR) | NR  (11-30) | NR  (5-30) | NR | 24 | Regression |
| Lawton, [29] | Joint Programme Neurodegenerative Disease, NR | PD | 1,161 | NR | 27.6± 2.3 (13-30) | 25.0± 3.5 (8-30) | Yes | 24 | Equipercentile equating with log-linear smoothing |
| Roalf, [16] | Penn Memory Center and University of Pennsylvania ADC, USA | CN, MCI, AD | 321 | 73.06±8.51 (50-93) | 24.78±3.40 (NR) | 20.27±4.39 (NR) | NR | 22 | Equipercentile equating with log-linear smoothing |
| Saczynski, [19] | General or geriatric medicine, large hospital, USA | CN, MCI, dementia | 119 | 84±5 (NR) | 24±5.8  (2-30) | 19±6.6  (0-30) | Yes | 22, 23 | Equipercentile equating |
| Solomon, [36] | Private memory clinic, USA | Probable AD, MCI due to AD | 101 | NR | NR | NR | NR | 23 | Linear regression |
| van Steenoven, [27] | Parkinson’s Disease and Movement Disorders Center, University of Pennsylvania and Parkinson’s Disease Research, Education and Clinical Center, Philadelphia VA Medical Center, USA | Idiopathic PD | 197 | 67.1±9.5 (NR) | 27.7 ±2.7 (12-30) | 24.7±4.2 (10-30) | Yes | 23 | Equipercentile equating with log-linear smoothing |
| Trzepacz, [17] | ADNI-GO, ADNI-2, USA | CN, MCI, AD | 618 | 76.49± 7.26  (56-94) | 25.74±2.62 (7-30) | 21.43±3.88 (1-30) | Yes | 21, 22 | Equipercentile equating with log-linear smoothing |
| Wong, [30] | CU-STRIDE study, Hong Kong | Stroke or TIA, CN, MCI, dementia | 623 | 68.7±10.9 (NR) | 25.0±4.6 (NR) | 19.8±6.2 (NR) | NR | 22 | Equipercentile equating with log-linear smoothing |
| Yu, [37] | PD study Dementia registry, Taiwan | Idiopathic PD without dementia | 168 | 69.67±9.48 (NR) | 25.54±3.76 (NR) | 21.83±4.99 (NR) | NR | 23 | Simple logistic regression |
| MMSE=Mini-Mental State Examination; MoCA-30=Montreal Cognitive Assessment administered in-person with a maximum possible score of 30; CN=cognitively normal; AD=Alzheimer’s disease; MCI=Mild Cognitive Impairment; VaD=Vascular Dementia; FTD=Fronto-Temporal Dementia; PPA=Primary Progressive Aphasia; PDD=Parkinson’s Disease Dementia; PSP=Progressive Supranuclear Palsy; PD=Parkinson’s Disease; NIA-AA=National Institute on Aging – Alzheimer’s Association; TIA=Transient Ischemic Attack; NR=not reported. | | | | | | | | | |

| **Supplementary table 5** Studies that provide MoCA-22 cutoffs for cognitive impairment in older adults | | | | | | | | |
| --- | --- | --- | --- | --- | --- | --- | --- | --- |
| First author, [reference] | Cohort and country | Diagnoses | Sample size | Age: mean±SD | MoCA-22 mode of administration | MoCA-22 score  mean±SD | MoCA-22 score adjusted for education | MoCA-22  cutoff  sensitivity specificity |
| Benge, [12] | Movement disorders center and support groups, USA | CN, MCI in Parkinson’s disease | 21 | 72.2±5.8 | Telephone | 18.5±2.0 | No | cutoff: 17  0.6  0.9 |
| Lai, [11] | Tertiary medical center, China | CN, MCI in atrial fibrillation | 101 | 62.1±8.1 | Telephone | NR | NR | cutoff: 17  85.7  69.7 |
| Pendlebury, [10] | Oxford Vascular Study, UK | MCI in post-acute vascular event | 68 | 73.4±7.0 | Telephone | 16.8±3.6 | No | cutoff: 19  89  46  cutoff: 18  81  59 |
| Wittich, [8] | Memory Clinics, Canada | CN, MCI, AD | 277 | 74.92±7.38 | Derived from MoCA-30 | NR | Yes | cutoff: 18  44  98  cutoff: 19  63  98 |
| Zietemann, [9] | Determinants of Dementia after Stroke, Germany | CN, MCI | 96 | 69.0±8.9 | Telephone | NR | Yes | cutoff: 19  81  73 |
| MoCA-22=Version of Montreal Cognitive Assessment that excludes items that require visual input and has a maximum possible score of 22; CN=cognitively normal; MCI=Mild Cognitive Impairment; AD=Alzheimer’s disease; NR=not reported. | | | | | | | | |
